# Supplementary material for: Intelligent single-shot full-field characterization over femtosecond pulses
Source: Nat Commun. 2025 Nov 25;16:11621. doi: 10.1038/s41467-025-66631-w (PMC12749541; doi:10.1038/s41467-025-66631-w)
Supplement: Supplementary file 1 — Supplementary Information [file 41467_2025_66631_MOESM1_ESM.pdf]

Intelligent single-shot full-field characterization over femtosecond pulses

Guoqing Pu*, Chao Luo, Weisheng Hu, and Lilin Yi*

State Key Laboratory of Photonics and Communications, School of Information Science and Electronic Engineering, Shanghai Jiao Tong University, Shanghai 200240, China

*Correspondence: teddyghf1994@sjtu.edu.cn, lilinyi@sjtu.edu.cn

A. Numerical simulations of dependences on sampling rates and noises.

The dependence on sampling rates of the acquisition device is studied in numerical simulations, and the results are summarized in Supplementary Fig. 1a. The single-shot pulse reconstruction performance of ISFC remains almost the same when the sampling rate is above the Nyquist sampling rate of ~19.7 GSa/s, corresponding to a shear value of ~9.85 GHz. The sampling rate of 12.5 GSa/s is completely acceptable for ISFC, as the duration NMAE of ~0.015 and the phase NRMSE of ~0.11. Further reducing the sampling rate to 6.25 GSa/s leads to sharp deterioration of the pulse reconstruction performance, as evidenced by the drastic increment of the phase NRMSE. An empirical deduction drawn from the simulation and experimental results indicates that the minimal sampling rate supported by ISFC lies between the actual shear value and its corresponding Nyquist sampling rate. Though we do not suppose ISFC violates the Nyquist sampling theorem. Because the shear value remains unchanged in simulations and it can be learnt by ISFC through numerous data. When the sampling rate is below the Nyquist sampling rate, ISFC automatically pads the missing but deterministic data points during computation. The shear value in experiments varies due to the environmental disturbances. However, the variation is rather small as the fringe frequencies of different temporal interferograms are all close to the calibrated shear value of ~2.13 GHz. As a result, the pulse reconstruction performance of ISFC is acceptable under the sampling rate of 3.13 GSa/s. On the other hand, the performance drop induced by using the sampling rate below the Nyquist sampling rate is quite obvious in both simulations and experiments. ISFC distinctly outperforms FTM under all tested sampling rates. Moreover, FTM is more dependent on higher sampling rates than ISFC. As the sampling rate reduces to 25 GSa/s, the duration retrieval accuracy of FTM experiences a substantial loss.

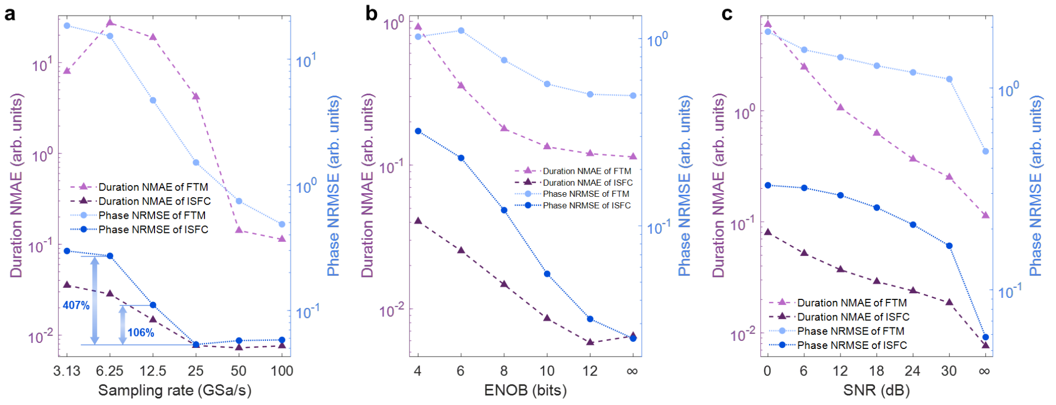

Supplementary Fig. 1 | Dependences on sampling rates and noises of ISFC and FTM in numerical simulations. a, Dependences on sampling rates, ISFC performs well when the sampling rate is above 12.5 GSa/s. **b**, Dependences on quantization noise, different levels of quantization noise are characterized by different ENOB. **c**, Dependences on random noises, different levels of random noise are characterized by different SNR.

Noise is an imperative factor in linear systems, and we quantitatively analyze the influence of noise in

numerical simulations. We categorize noises into two types: quantization noise introduced in digitalization and other noises (e.g., shot noise, thermal noise) modelled by Gaussian white noise in simulations. The effective number of bits (ENOB) is used as a metric to evaluate the quantization noise level, and there is no quantization when ENOB is infinite. As shown in Supplementary Fig. 1b, ISFC performs significantly better than FTM under different ENOB. When gradually reducing ENOB from 12 bits, the pulse reconstruction performance of both algorithms becomes worse. The performance of ISFC under 8-bit ENOB is decent with the duration NMAE of ~0.015 and the phase NRMSE of ~0.12. The real-time oscilloscope in experiments has a claimed resolution of 8 bits, thus, its ENOB is less than 8 bits. Therefore, quantization noise induced a pulse reconstruction performance drop of ISFC that is non-negligible in experiments.

To quantitatively evaluate the influence of other noises, different levels of Gaussian white noise characterized by signal-to-noise ratio (SNR) are added to temporal interferograms generated in numerical simulations. Supplementary Fig. 1c shows the pulse reconstruction performance of both algorithms under different SNR, and no noise is added when the SNR is infinite. Again, ISFC significantly outperforms FTM. A distinct performance drop of both algorithms is observed when random noise is first introduced. Under 30-dB SNR, the duration NMAE of ~0.019 and the phase NRMSE of ~0.17 for ISFC. Then, pulse reconstruction performance gradually decreases as noise becomes stronger. Hence, various noises (e.g., shot noise, thermal noise) in experiments substantially account for the pulse reconstruction performance discrepancies of ISFC in simulations and experiments. Last but not least, TOD is ignored in all datasets used for analyzing dependences on sampling rates and noises. The superiority of ISFC will be further magnified when TOD-induced nonlinear spectral-to-temporal mapping is considered.

B. Calibration over the delay and dispersion values in experiments

To apply FTM in experiments, the spectral shear created in LSSI must be calibrated for non-iterative phase retrieval. Therefore, we need to calibrate the delay of the Mach-Zehnder interferometer and the dispersion values of DCF in LSSI. The delay is measured via white-light interferometry, where spectral fringes with a period of $\Delta\upsilon = 1/\Delta t$ can be observed[1]. In experiments, an amplified spontaneous emission source is used as the white-light source, and the spectral interferogram is shown in Supplementary Fig. 2. By performing inverse Fourier transform on the spectral interferogram, the delay is the temporal position corresponding to the peak of either sideband, and the measured delay is ~5.8 ps.

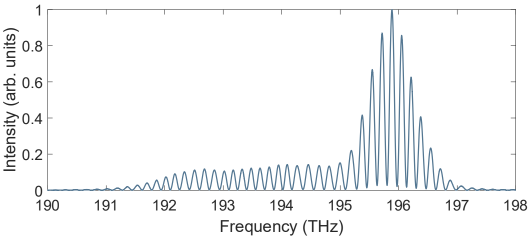

Supplementary Fig. 2 | Spectral interferogram obtained with an amplified spontaneous emission source for delay calibration.

On the other hand, dispersion values are calibrated via the spectral-to-temporal mapping built on DFT, which can be expressed as[2]

$$f = f_0 + \frac{1}{2\pi\beta_2 L}t - \sum_{k=3}^{\infty}\frac{\beta_k L}{2\pi(k-1)!(\beta_2 L)^k}t^{k-1}. \qquad (S1)$$

f_0 is the centre frequency, β_k is the k-th order coefficient of the propagation constant, and L is the length of the dispersive element. We first create a near-Fourier-transform-limited pulse with the programmable spectral filter.

Then, its optical spectrum before DFT (i.e., the red curve at the top of Supplementary Fig. 3) and temporal interferograms are recorded. 161 consecutive temporal frames are obtained, and the coherent average frame (i.e., the black curve in the bottom of Supplementary Fig. 3) is calculated by aligning the 161 segmented frames (i.e., grey curves in the bottom of Supplementary Fig. 3) with cross correlation. Spectral peaks and the corresponding temporal peaks of the coherent average frame are selected as shown in Supplementary Fig. 3. The spectral and temporal coordinates corresponding to the spectral and temporal peaks should satisfy Equation S1. Thus, the dispersion parameters can be readily obtained through polynomial fitting between the spectral coordinates and temporal coordinates. The group velocity dispersion of the DCF in experiments is calibrated to be ~-339.65 ps/nm, which is close to the claimed group velocity dispersion of -340 ps/nm, proving the validity of the dispersion calibration. The TOD and fourth-order dispersion are calibrated to be ~-1.89 ps^3 and ~-0.49 ps^4, respectively.

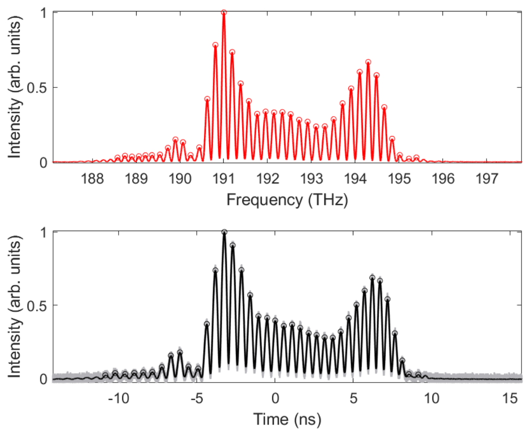

Supplementary Fig. 3 | Spectral interferogram and temporal interferograms obtained with a near-Fourier-transform-limited pulse for dispersion calibration. Top, Spectral interferogram before DFT; **Bottom**, 161 segmented temporal frames (grey) and their coherent average result (black). The circles indicate selected spectral and temporal peaks for dispersion calibration.

C. Another switching dynamics of the programmable spectral filter resolved by ISFC in single shot

Supplementary Fig. 4 shows another switching dynamics of the programmable spectral filter resolved by ISFC in single shot under the sampling rate of 3.13 GSa/s. As the temporal intensity dynamics shown in the left subplot of Supplementary Fig. 4, the switching starts from the frame ~1.7 million and finishes at the frame ~3.8 million, corresponding to the switching time of 64.8 ms. A small peak originates on the left side during the switching at the frame ~2.4 million, and it gradually merges with the main peak as the switching proceeds. The reconstructed pulses by ISFC of the start frame, 3 middle frames, and the final frame are shown in Supplementary Fig. 4b. Again, the reconstructed start and final pulses are consistent with the FROG measurements in both temporal and spectral domains.

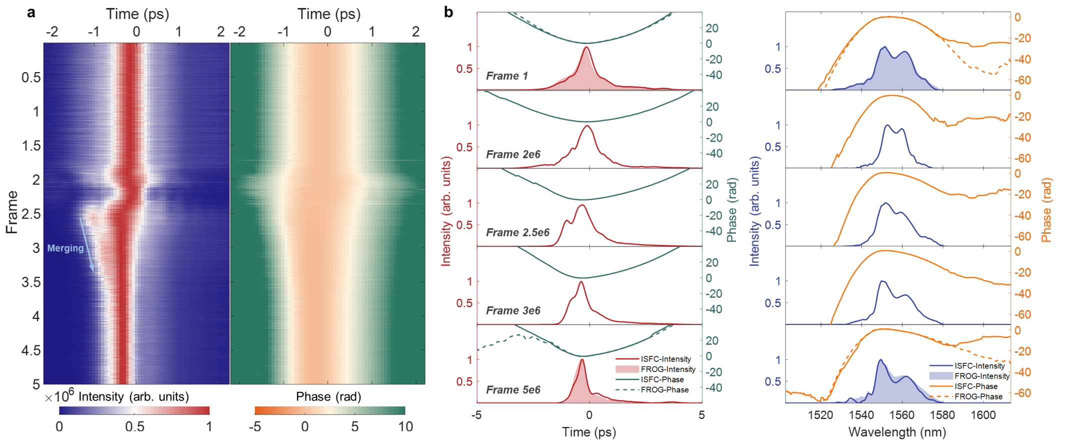

Supplementary Fig. 4 | Another switching dynamics of the programmable spectral filter resolved by ISFC in single shot. a, The intensity (left) and phase (right) evolution dynamics in a switching process of the programmable spectral filter resolved by ISFC in single shot under the sampling rate of 3.13 GSa/s. **b**, Reconstructed pulses of the start frame, 3 middle frames, and the final frame, where the reconstructed start and final pulses align well with the FROG measurements.

D. Characterization over shorter fs pulses with LSSI-based ISFC

To validate the ability of the proposed LSSI-based ISFC in even shorter fs pulses characterization, we generate a shorter-fs-pulse dataset in numerical simulations, which contains 60000 pulses with durations ranging from 8 fs to 50 fs. Due to the wide bandwidth, the fibre length for performing DFT can be reduced to 100 m to shorten the simulation window to 6 ns, and TOD of the fibre is considered. The time delay of the interferometer is fixed at 1 ps thereby corresponding to the shear frequency of ~59.12 GHz. In the dataset, 54000 pulses are used for training, and the rest 6000 pulses are used for testing. We train the ISFC under the sampling rate of 100 GSa/s and the shorter fs pulse reconstruction results are shown in Supplementary Fig. 5. The average duration MAE and the average phase RMSE over 6000 pulses in the test set are 0.01 fs and 0.071 rad respectively, manifest rather good pulse reconstruction.

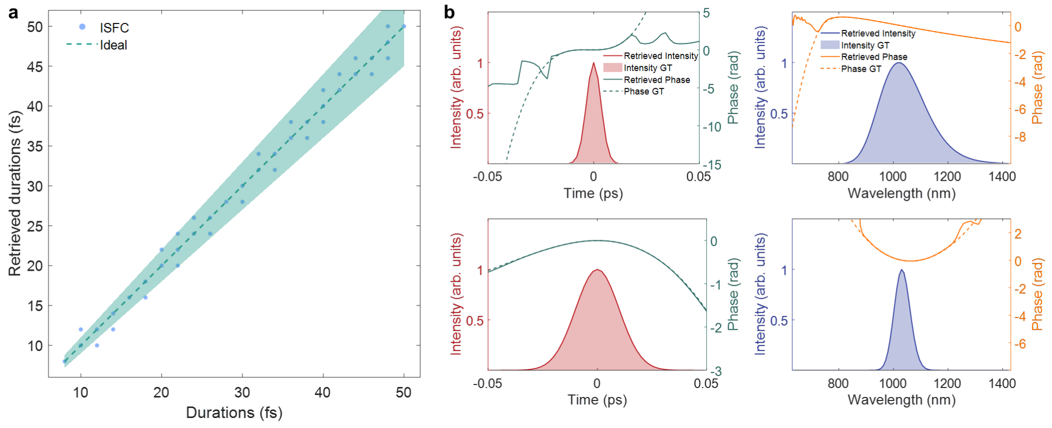

Supplementary Fig. 5 | Characterization over shorter fs pulses with LSSI-based ISFC. a, Pulse duration retrieval results of ISFC over the dataset with TOD, where the dashed line and shadow area respectively denote the ideal situation and the 10% error range. **b**, Temporal retrieval results (the left column) of ISFC over an 8-fs pulse and a 20-fs pulse from the test set, and corresponding spectral retrieval results (the right column) via performing Fourier transform on the retrieved temporal pulses.

Supplementary References

1. Reynaud F, Salin F, Barthelemy A. Measurement of phase shifts introduced by nonlinear optical phenomena on subpicosecond

pulses. Optics Letters, 1989, 14(5): 275-277.

2. Xia H, Yao J. Characterization of subpicosecond pulses based on temporal interferometry with real-time tracking of higher order dispersion and optical time delay. Journal of Lightwave Technology, 2009, 27(22): 5029-5037.
